# Causal effect of early life adiposity on gestational diabetes mellitus and mediating roles of lipidomic biomarkers

**DOI:** 10.3389/fnut.2023.1225376

**Published:** 2023-07-19

**Authors:** Chuang Li, Na Li, Caixia Liu, Huan Li

**Affiliations:** ^1^Department of Obstetrics & Gynecology, Shengjing Hospital of China Medical University, Shenyang, Liaoning, China; ^2^Key Laboratory of Maternal-Fetal Medicine of Liaoning Province, Shenyang, Liaoning, China

**Keywords:** early life, obesity, gestational diabetes mellitus, lipidomic biomarkers, Mendelian randomization

## Abstract

**Objective:**

The causal relationship between early life adiposity and gestational diabetes mellitus (GDM) and the underlying mechanisms remains unclear. This study aimed to investigate the independent causal association between early life adiposity and GDM and identify potential metabolic mediators and their mediating effects on this relationship.

**Methods:**

Using genome-wide association study (GWAS) summary statistics from the publicly available database of early life adiposity (5,530 cases and 8,318 controls) and GDM (11,279 cases and 179,600 controls), a two-step, two-sample Mendelian randomization (MR) was conducted to estimate the causal mediation effects of lipidomic biomarkers including low-density lipoprotein cholesterol (LDL-C), high-density lipoprotein cholesterol (HDL-C), triglyceride, apolipoprotein A-Ι, and apolipoprotein B on the relationship between early life adiposity and GDM.

**Results:**

Genetically predicted childhood adiposity was positively associated with risk of GDM (OR: 1.21, 95%CI: 1.09–1.34, *p* = 4.58 × 10^−4^). This causal relationship remained after accounting for adult adiposity traits in the multivariable MR analyses. Two-step MR identified three candidate mediators that partially mediated the effect of early life adiposity on GDM, including HDL-C (5.81, 95%CI: 3.05–8.57%), apolipoprotein A-Ι (4.16, 95%CI: 1.64–6.69%), and triglyceride (2.20, 95%CI: 0.48–3.92%).

**Conclusion:**

This MR study demonstrated that the causal effect of childhood obesity on future GDM risk was independent of adult adiposity. We identified three mediators, including HDL-C, apolipoprotein A-Ι, and triglyceride, in this association pathway. Our results provide insights into the pathogenesis of GDM and suggest additional prevention and treatment targets for GDM related to early life adiposity.

## 1. Introduction

Gestational diabetes mellitus (GDM) is hyperglycemia that develops during pregnancy and usually resolves after birth (1). GDM affects up to 30% of pregnant women worldwide depending on the population, screening method, and diagnostic criteria (1). GDM has long been linked to adverse obstetric and neonatal outcomes and is mainly associated with higher infant birth weight (2). Moreover, GDM has been recognized as a risk factor for future cardiometabolic diseases in mothers and offspring (3). Observational and interventional studies have identified several modifiable and nonmodifiable risk factors for GDM, including advanced maternal age, family history of diabetes, previous GDM, previous macrosomia, overweight/obesity, and cigarette smoking, some of which are targeted in preventive and therapeutic strategies (4–7).

Childhood obesity poses a major threat to global public health, with an increasing prevalence in most parts of the world over the past decades (8). Irrespective of adiposity later in life, increasing childhood obesity may have significant consequences for population health, given evidence from observational studies and Mendelian randomization (MR) studies linking early life excess body weight to higher risks of chronic diseases, including hypertension and type 2 diabetes (9–11). Thus, childhood and adolescence may be critical periods during which adiposity affects the risk of developing metabolic disorders in adulthood. However, few studies have examined the impact of childhood obesity on the risk of developing GDM. Thus, whether childhood obesity contributes to the development of GDM later in life remains unclear.

MR uses genetic variation as an instrumental variable (IV) to estimate the causal association between exposure and outcome (12). Compared with traditional observational analyses, MR analyses are less prone to confounding and reverse causation, given the random allocation and fixed nature of genetic variants (13). Univariate MR (UVMR) analysis can estimate the total effect of early life adiposity on GDM risk (14). Moreover, multivariable MR (MVMR) allows the estimation of the independent effects of childhood obesity on GDM risk, independent of adult body size (15–17). It can also be used to examine the mediation between early life adiposity and GDM (18).

In this study, we conducted a two-sample MR analysis to examine the independent causal association between childhood obesity and GDM risk. Furthermore, we used two-step MR to investigate the potential mediators and quantify their mediating effects on this relationship.

## 2. Materials and methods

### 2.1. Study design

MR was conducted in two stages. In stage 1, we performed a two-sample UVMR using summary-level data to assess the causal effect of childhood obesity on the risk of GDM. We then used MVMR to estimate the independent effect of childhood obesity on GDM after accounting for adult adiposity measures. In stage 2, we first screened for lipid traits, including low-density lipoprotein cholesterol (LDL-C), high-density lipoprotein cholesterol (HDL-C), triglyceride, apolipoprotein A-Ι, and apolipoprotein B, as potential mediators of the association between childhood obesity and GDM. We then performed a two-step MR to evaluate the mediation effect of each selected mediator on the causal relationship between childhood obesity and GDM.

### 2.2. Data sources

#### 2.2.1. Early and later life adiposity traits

We obtained genome-wide association study (GWAS) summary statistics of childhood obesity from the Early Growth Genetics (EGG) Consortium (19). The GWAS meta-analysis consisted of 14 relevant studies, with 5,530 cases (≥95% body mass index [BMI] reached before the age of 18 years) and 8,318 controls (<50% BMI consistent throughout all measures during childhood) (Table 1). The childhood BMI was calculated from the height and weight measurements obtained at ages 2–18 years, except for Avon Longitudinal Study of Parents and Children (ALSPAC), which leveraged BMI data available from the first four clinical examinations prior to 2 years old. The summary-level data for adult BMI were accessed from a GWAS meta-analysis, which included association results for up to from 125 studies, 82 with GWAS results (*n* = 236,231) and 43 with results from Metabochip (*n* = 103,047) (20). The GWAS summary statistics for adult waist circumference (WC) and waist-to-hip ratio (WHR) were obtained from the previously described meta-analysis, which included 142,762 individuals of European ancestry from 57 cohorts genotyped with GWAS and 67,326 individuals from 44 cohorts genotyped with the Metabochip (21) (Table 1).

**Table 1:** GWAS Data sources of the MR study.

Phenotype	Data type	Sample size	Population	Consortium/cohort
Exposure				
Childhood obesity	Continuous	766,345	European	EGG
Adult adiposity traits				
BMI (adult)	Continuous	339,224	Mixed	GIANT
WC (adult)	Continuous	231,353	European	GIANT
WHR (adult)	Continuous	212,244	European	GIANT
Outcome				
GDM	Continuous	190,879	European	FinnGen
Lipid traits				
LDL-C	Continuous	440,546	European	UK Biobank
HDL-C	Continuous	403,943	European	UK Biobank
Triglycerides	Continuous	441,016	European	UK Biobank
Apolipoprotein A-Ι	Continuous	393,193	European	UK Biobank
Apolipoprotein B	Continuous	439,214	European	UK Biobank

GDM, gestational diabetes mellitus; BMI, body mass index; WC, waist circumference; WHR, waist-hip ratio; LDL-C, low-density lipoprotein cholesterol; HDL-C, high-density lipoprotein cholesterol.

#### 2.2.2. Lipid traits

For lipid traits, we obtained summary statistics for LDL-C, HDL-C, triglyceride, apolipoprotein A-Ι, and apolipoprotein B from the GWAS data provided by the UK Biobank (22) (Table 1). The GWAS of lipids and apolipoproteins in the UK biobank included a sample size ranging between 393,193 and 441,016 individuals, with a mean age of 56.9 years and a female representation of 54.2%.

#### 2.2.3. GDM

Summary statistics for GDM were obtained from Release 8 results of GWAS data from the FinnGen consortium. This GWAS data included 11,279 GDM cases (identified using registry data on the International Classification of Diseases [ICD] 9 and 10 codes O24.4) with a mean age of 31.2 years (23) (Table 1).

Details of the recruitment, information on genetic data, and measurements of baseline characteristics of each cohort are obtained from the original study.

### 2.3. Selection of genetic IVs

To obtain reliable IVs, three key assumptions of MR must be satisfied (24). First, IVs are strongly associated with early life adiposity/lipid traits. Second, IVs are independent of confounders of the exposure-outcome relationship (excluding mediators). Third, IVs affect the outcome only through exposure and mediators, and not through any other paths.

We screened for genetic variants with genome-wide significance (*p* < 5 × 10^−8^) and linkage disequilibrium (*r*^2^ < 0.001) within a 10,000 kb window (25). Palindromic single-nucleotide polymorphisms (SNPs) with incompatible alleles were also removed. Proxy SNPs were used when the SNPs were unavailable in the GWAS outcome data. The final IVs for subsequent MR studies consisted of rigorously selected SNPs. To avoid a weak IVs bias, the *F*-statistic was calculated to evaluate the strength of the selected IVs (26).

### 2.4. Statistical analyses

#### 2.4.1. UVMR and MVMR analyses

For UVMR analyses, the inverse variance-weighted (IVW) method was used as the main analysis (13). The MR-Egger (27), weighted median (28), and weighted mode methods (29) were used as sensitivity analyses to assess the robustness of the IVW estimate. We performed MR pleiotropy residual sum and outliers (MR-PRESSO) to detect outliers with horizontal pleiotropy among the chosen SNPs (30). We evaluated heterogeneity using Cochran’s Q statistic and identified horizontal pleiotropy based on the MR-Egger regression model intercept (27, 31).

For MVMR analyses, the multivariate inverse variance-weighted (MV-IVW) method was used as the main analysis. The multivariate MR Egger (MVMR-Egger) method was used for sensitivity analysis.

To control for false positive rates, we used a Bonferroni-corrected threshold of *p* < 0.01 (*α* = 0.05/5) when analyzing the causal effects of childhood obesity on lipid traits, lipid traits on GDM, and lipid traits on childhood obesity.

#### 2.4.2. Two-step MR analyses

We then conducted two-step MR analyses to assess and quantify the mediating effect of the selected mediators on the causal relationship between childhood obesity and GDM. The first step was to estimate the causal effect (β1) of childhood obesity on each chosen mediator using a UVMR analysis. The second step estimated the causal effect (β2) of each mediator on GDM risk, adjusted for childhood obesity, using an MVMR analysis. We calculated each mediator’s proportion of the total effect of childhood obesity on GDM by dividing the mediation effect (β1 × β2) by the total effect (32). We derived the standard errors for the mediation effects using the delta method (33).

All analyses were performed using R (Version 4.1.3) with the R package “TwosampleMR,” “Mendelian Randomization,” and “MR-PRESSO” (34, 35).

## 3. Results

### 3.1. Causal effect of childhood obesity on GDM

**Figure 1:**
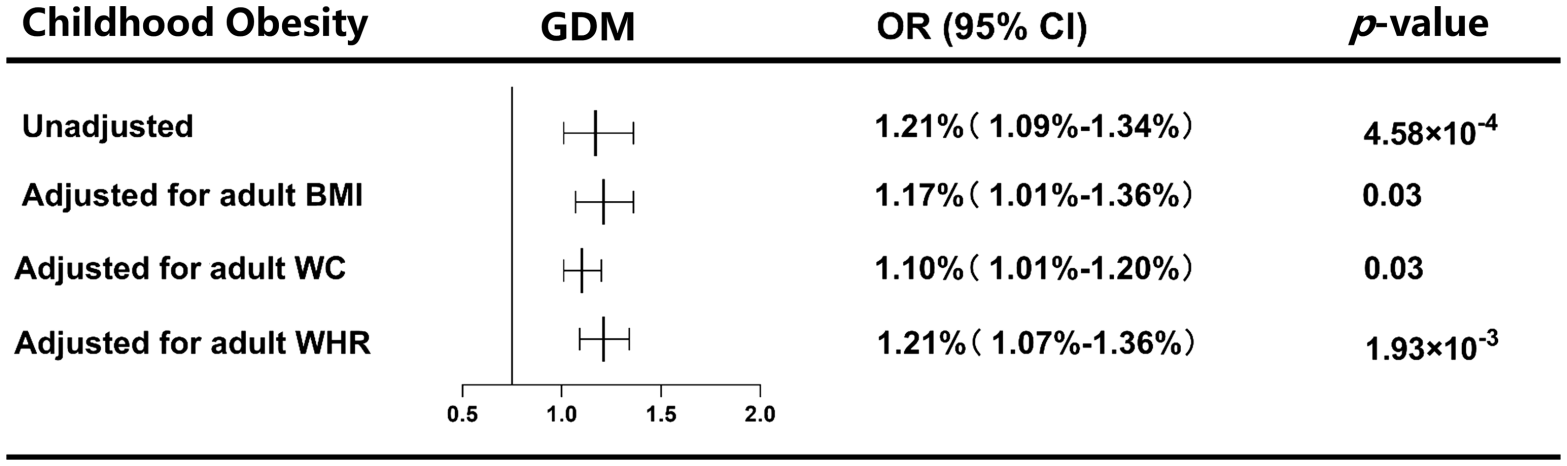
UVMR and MVMR estimates for the causal, independent effect of childhood obesity on GDM risk.

In UVMR, genetically predicted childhood obesity was positively associated with GDM risk (OR: 1.21, 95%CI: 1.09–1.34, *p* = 4.58 × 10^−4^) (Figure 1). Sensitivity analyses using weighted median (OR: 1.41, 95%CI: 1.11–1.36, *p* = 9.81 × 10^−4^), weighted mode (OR: 1.24, 95%CI: 1.10–1.41, *p* = 0.03), and MR-PRESSO methods (OR: 1.21, 95%CI: 1.09–1.34, *p* = 0.02) supported the robustness of the IVW method (Supplementary Table S1). MR-PRESSO did not detect any outlier SNPs. The MR-Egger intercept test showed no evidence of pleiotropy (*p*_intercept_ = 0.76). Cochran’s Q statistic indicated no potential heterogeneity among the selected SNPs (*p*_heterogeneity_ = 0.07). The mean *F*-statistic for the selected IVs was 45, indicating that the estimates did not suffer from weak instrumental bias.

In MVMR analyses, the causal effect of childhood obesity on GDM remained significant after accounting for adult BMI (OR: 1.17, 95%CI: 1.01–1.36, *p* = 0.03), WC (OR: 1.10, 95%CI: 1.01–1.20, *p* = 0.03), or WHR (OR: 1.21, 95%CI: 1.07–1.36, *p* = 1.94 × 10^−3^) (Figure 1). Sensitivity analyses using the MVMR-Egger method further confirmed the robustness of the MV-IVW method (Supplementary Table S2).

### 3.2. Causal effect of child obesity on lipid traits

The IVW results suggested that childhood obesity was negatively associated with the genetically determined level of LDL-C (β: -0.02, 95CI%: −0.03 – −0.004, *p* = 7.49 × 10^−3^), HDL-C (β: −0.04, 95CI%: −0.05 – −0.03, *p* = 1.35 × 10^−7^), and apolipoprotein A-Ι (β: −0.04, 95CI%: −0.05 – −0.02, *p* = 2.28 × 10^−5^) and was positively associated with the genetically determined level of triglyceride (β: 0.02, 95CI%: 0.005–0.03, *p* = 5.29 × 10^−3^) (Table 2). The mean *F*-statistics for the genetic instruments were greater than 10, indicating limited weak instrument bias (Table 2). The MR-Egger intercept test indicated no evidence of horizontal pleiotropy between childhood obesity and the selected mediators (Supplementary Table S3). Moreover, Cochran’s Q statistics showed no significant evidence of heterogeneity among the IVs (Supplementary Table S3).

**Table 2:** UVMR association of childhood obesity with each lipid trait from the IVW results.

Lipid traits	No. SNP	*F*-statistics	Beta	SE	95%CI	*p-*value
LDL-C	4	42	−0.02	0.006	−0.03 – −4.0 × 10^−3^	7.49 × 10^−3^
HDL-C	2	45	−0.04	0.008	−0.05 – −0.03	1.35 × 10^−7^
Triglyceride	4	46	0.02	0.006	4.62 × 10^−3^ – 0.03	5.29 × 10^−3^
Apolipoprotein A-Ι	2	45	−0.04	0.008	−0.05 – −0.02	2.28 × 10^−5^
Apolipoprotein B	4	42	7.33 × 10^−3^	0.006	−0.02 – 4.55 × 10^−3^	0.23

### 3.3. Causal effect of lipid traits on GDM adjusted for childhood obesity

MVMR analyses indicated that the causal effect of HDL-C (OR: 0.76, 95%CI: 0.65–0.89, *p* = 7.36 × 10^−4^), triglyceride (OR: 1.30, 95%CI: 1.11–1.53, *p* = 1.49 × 10^−3^), and apolipoprotein A-Ι (OR: 0.75, 95%CI: 0.63–0.89, *p* = 8.66 × 10^−4^) on GDM remained significant after accounting for childhood obesity (Table 3). The MVMR-Egger sensitivity analysis confirmed the robustness of the MV-IVW method (Supplementary Table S4).

**Table 3:** MVMR association of lipid traits with GDM risk adjust for childhood obesity.

Lipid traits	Beta	SE	OR	95%CI	*p-*value
LDL-C	−0.13	0.17	0.88	0.63–1.24	0.47
HDL-C	−0.27	0.08	0.76	0.65–0.89	7.36 × 10^−4^
Triglyceride	0.26	0.08	1.30	1.11–1.53	1.49 × 10^−3^
Apolipoprotein A-Ι	−0.29	0.09	0.75	0.63–0.89	8.66 × 10^−4^
Apolipoprotein B	−0.13	0.17	0.88	0.63–1.24	0.47

### 3.4. Mediation effect of potential mediators

**Figure 2:**
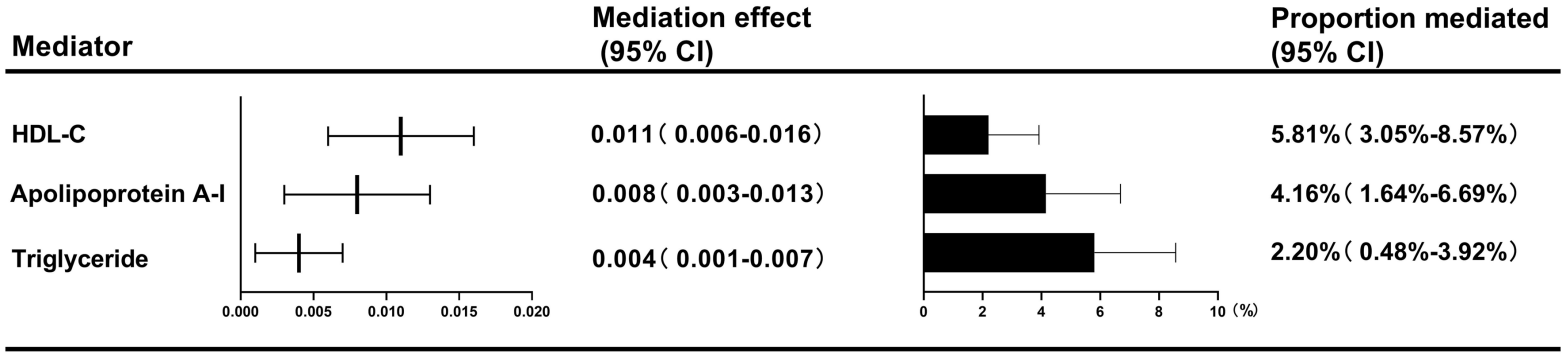
Two-step MR estimates for the causal influence of childhood obesity on GDM risk by each potential mediator.

Two-step MR analyses indicated three potential mediators, including HDL-C, apolipoprotein, and triglyceride, which might be responsible for the causal effect of childhood obesity on GDM risk. HDL-C mediated the total effect of childhood obesity on GDM risk (5.81, 95%CI: 3.05–8.57%), followed by apolipoprotein A-Ι (4.16, 95%CI: 1.64–6.69%), and triglyceride (2.20, 95%CI: 0.48–3.92%) (Figure 2).

## 4. Discussion

In this MR study, we utilized two-sample and two-step MR approaches, for the first time, to investigate the causal associations between childhood obesity and GDM. Notably, we comprehensively explored the roles of lipid profiles in mediating the relationships of childhood obesity with GDM risk. Our main findings were threefold. First, we demonstrated that genetically predicted childhood obesity increased the risk of GDM, and this deleterious effect persisted after adjusting for adult adiposity traits, including BMI, WC, and WHR. Second, we found that childhood obesity was causally related to decreased levels of LDL-C, HDL-C, and apolipoprotein A-Ι and increased levels of triglyceride, while HDL-C, apolipoprotein A-Ι, and triglyceride were further causally associated with risk of GDM. Third, we identified HDL-C, apolipoprotein A-Ι, and triglyceride as potential mediators of the causal effect of childhood obesity on GDM.

Previous studies have identified a causal role of childhood obesity in the development of multiple chronic diseases, including hypertension and type 2 diabetes. However, few studies have focused on the association between childhood obesity and GDM. A retrospective study of 13,031 women with anthropometric information measured during childhood showed that a higher childhood BMI was associated with an increased risk of GDM (36). A cohort of 1,386 GDM patients found no association between heavy body shape at 10 years of age and the risk of self-reported GDM (37). A longitudinal study also showed that mean childhood BMI was not associated with the risk of GDM (38). The influence of potential confounders such as adult adiposity may partly explain the conflicting observations in existing studies. Using MR approaches to minimize potential confounding factors, our study built on previous evidence by demonstrating that childhood obesity causally and adversely affects GDM independently of adult adiposity. These findings highlight the significance of childhood obesity as a key indicator in GDM risk prediction and prevention.

To identify the biochemical mechanisms through which childhood obesity influences the risk of GDM, we further explored whether there are causal mediators of subsequent life trajectories that modulate the relationship between childhood obesity and GDM. We identified HDL-C, apolipoprotein A-Ι, and triglyceride levels as causal mediators of the impact of childhood obesity on GDM. Our findings are in line with the results of previous studies using metabolomic and lipidomic approaches, which showed that altered lipid traits associated with insulin resistance, inflammation regulation, and oxidative stress were involved in the pathophysiology of GDM (39, 40). However, further studies are required to confirm the mechanisms underlying childhood obesity-related GDM.

This study had several strengths. First, we designed a rigorous MR framework to establish causality between childhood obesity and GDM as well as mediation mechanisms. Second, we employed multiple complementary sensitivity analyses to verify the reliability of the MR findings. Third, we used large-scale GWAS summary statistics, which increased the statistical power and accuracy of the causal effect estimates.

This study also had several limitations. First, we assumed that the associations between childhood obesity and GDM were linear in both UVMR and MVMR analyses. Further research utilizing individual-level data is warranted to examine the potential nonlinear causal connections between childhood obesity and GDM. Second, we concentrated on the lipid traits that were theoretically linked to childhood obesity or GDM as candidate mediators. However, the mechanisms connecting childhood obesity and GDM were not fully elucidated.

In conclusion, this MR study supports the causal effect of childhood obesity on GDM risk. Furthermore, our findings suggest that this association is partially mediated by the lipid traits, HDL-C, apolipoprotein, and triglyceride. Understanding the causal relationships between childhood obesity, dyslipidemia, and GDM is crucial for elucidating the pathogenesis of GDM and identifying potential targets for early intervention.

## Data availability statement

The original contributions presented in the study are included in the article/Supplementary material, further inquiries can be directed to the corresponding author.

## Ethics statement

The studies involving human participants were reviewed and approved by Ethics committee of Shengjing Hospital of China Medical University. Written informed consent for participation was not required for this study in accordance with the national legislation and the institutional requirements. The animal study was reviewed and approved by Ethics committee of Shengjing Hospital of China Medical University.

## Author contributions

CXL and HL conceptualized and designed the study. CL and NL performed the analyses and interpretation of data. CL wrote the first draft of the manuscript. CL, CXL, and HL contributed to manuscript review and editing, and read. All authors contributed to the article and approved the submitted version.
