# Supplementary material for: Causal effect of early life adiposity on gestational diabetes mellitus and mediating roles of lipidomic biomarkers
Source: Front Nutr. 2023 Jul 19;10:1225376. doi: 10.3389/fnut.2023.1225376 (PMC10394696; doi:10.3389/fnut.2023.1225376)
Supplement: Supplementary file 1 [file Data_Sheet_1.docx]

Supplementary Material

Causal Effect of Early Life Adiposity on Gestational Diabetes Mellitus and Mediating Roles of Lipidomic Biomarkers

**Chuang Li ^1,2^, Na Li^1,2^, Caixia Liu^1,2^, Huan Li ^1,2^**

**^1^ Department of Obstetrics & Gynecology, Shengjing Hospital of China Medical University, Shenyang 110004, Liaoning, China**

**^2^ Key Laboratory of Maternal-Fetal Medicine of Liaoning Province, Shenyang 110004, Liaoning, China
Corresponding Author
Huan Li, E-mail: lihuan7934sj@163.com**

| **Table S1 UVMR of the association between childhood obesity and GDM risk from the sensitivity analyses** | | | | | |
| --- | --- | --- | --- | --- | --- |
| **Methods** | **Beta** | **SE** | **OR** | **95%CI** | ***p-*value** |
| MR-Egger | 0.34 | 0.46 | 1.41 | 0.57–3.49 | 0.51 |
| Weighted median | 0.20 | 0.05 | 1.23 | 1.11–1.36 | 9.81×10^-5^ |
| Weighted mode | 0.22 | 0.06 | 1.24 | 1.10–1.41 | 0.03 |
| MR-PRESSO | 0.19 | 0.05 | 1.21 | 1.09–1.34 | 0.02 |

UVMR, univariate Mendelian randomization; GDM, gestational diabetes mellitus; MR- PRESSO, Mendelian randomization pleiotropy residual sum and outliers; OR, odds ratio; SE, standard error; CI, confidence interval

| **Table S2 MVMR association of childhood obesity with GDM risk adjusting for adult adiposity traits** | | | | | | | | |
| --- | --- | --- | --- | --- | --- | --- | --- | --- |
| **Model** | **MVMR Egger** | | | **Heterogeneity test** | | **Directional pleiotropy test** | | |
|  | **OR** | **95%CI** | ***p-*value** | **Q statistic** | ***p-*value** | **Egger intercept** | **SE** | ***p-*value** |
| Adjusted for BMI (adult) | 1.21 | 1.03–1.43 | 0.02 | 206.58 | 3.63×10^-15^ | -0.004 | 0.005 | 0.38 |
| Adjusted for WC (adult) | 1.18 | 1.05–1.32 | 4.67×10^-3^ | 114.11 | 6.24×10^-5^ | -0.006 | 0.004 | 0.07 |
| Adjusted for WHR (adult) | 1.27 | 1.10–1.48 | 1.50×10^-3^ | 61.13 | 1.87×10^-4^ | -0.007 | 0.006 | 0.26 |

MVMR, multivariate Mendelian randomization; GDM, gestational diabetes mellitus; BMI, body mass index; OR, odds ratio; CI, confidence interval; SE, standard error; WC, waist circumference; WHR, waist-to-hip ratio

| **Table S3 Heterogeneity and directional pleiotropy test of the association of childhood obesity with lipid traits** | | | | | | |
| --- | --- | --- | --- | --- | --- | --- |
| **Lipid traits** | **Heterogeneity test** | | | **Directional pleiotropy test** | | |
|  | **Q statistic** | **Q df** | **Q *p-*value** | **Egger intercept** | **SE** | ***p*-value** |
| LDL-C | 1.35 | 3 | 0.72 | -0.002 | 0.008 | 0.85 |
| HDL-C | 0.17 | 1 | 0.68 |  |  |  |
| Triglycerides | 0.98 | 3 | 0.81 | -0.005 | 0.007 | 0.59 |
| Apolipoprotein A-Ι | 1.16 | 1 | 0.28 |  |  |  |
| Apolipoprotein B | 0.83 | 3 | 0.84 | -0.005 | 0.008 | 0.58 |

SE, standard error; LDL-C, low-density lipoprotein cholesterol; HDL-C, high-density lipoprotein cholesterol

| **Table S4 MVMR association of lipid traits with GDM risk adjusting for childhood obesity** | | | | | | | | |
| --- | --- | --- | --- | --- | --- | --- | --- | --- |
|  |  |  |  |  |  |  |  |  |
| **Lipid traits** | **MVMR Egger** | | | **Heterogeneity test** | | **Directional pleiotropy test** | | |
|  | **OR** | **95%CI** | ***p-*value** | **Q statistic** | ***p-*value** | **Egger intercept** | **SE** | ***p-*value** |
| LDL-C | 0.71 | 0.53–0.94 | 0.02 | 124.89 | 1.49×10^-8^ | -0.007 | 0.005 | 0.16 |
| HDL-C | 0.75 | 0.65–0.88 | 4.50×10^-4^ | 196.26 | 1.21×10^-7^ | -0.004 | 0.003 | 0.21 |
| Triglycerides | 1.29 | 1.09–1.52 | 2.54×10^-3^ | 206.28 | 1.55×10^-9^ | -0.003 | 0.004 | 0.45 |
| Apolipoprotein A-Ι | 0.74 | 0.63–0.88 | 7.33×10-4 | 168.26 | 3.08×10^-8^ | -0.001 | 0.003 | 0.72 |
| Apolipoprotein B | 0.86 | 0.61–1.21 | 0.38 | 1.51E+02 | 2.52×10^-12^ | -0.005 | 0.005 | 0.35 |

MVMR, multivariate Mendelian randomization; GDM, gestational diabetes mellitus; OR, odds ratio; CI, confidence interval; SE, standard error; LDL-C, low-density lipoprotein cholesterol; HDL-C, high-density lipoprotein cholesterol
